# Food in War-Time

**Published:** 1918-04-13

**Authors:** 


### FOOD IN WAR-TIME.

### The Fish Dinner.

Fish dinners are seldom popular as presented in the average large establishment. Institution cooks have a sad habit of serving a flavourless substance, largely made up of skin and bones, under the guise of a fish dinner, or that is how it sometimes appears to the diner when the fish reaches his plate. We have been struck when serving dinners to wounded soldiers with the exceedingly unsubstantial nature of a helping of boiled fish. It comes into the room in the form of a handsome cod. Three or four watery ounces are dealt out to each, together with a spoonful of mashed potatoes. In three minutes every plate is empty, and quite obviously no one is conscious of having dined. Further helpings are a mere jumble of chilled fragments. Is this an exaggerated description?

The more delicate kinds of fish are now outrageously dear. In institutions where economy must be practised it is the commoner sorts of fish which must be procured, and these are even less satisfactory when boiled and served without the aid of rich sauces than cod or halibut. Moreover, much of the best nourishment contained in fish is boiled out when it is cooked in this easy but careless fashion. In order to secure that all the nourishing properties of the fish are preserved it should be cooked in a conservative fashion. To satisfy the appetite of the diner it must be accompanied by or served with some more satisfying substance, and in order to preserve it from insipidity some flavour must be introduced.

It is a good plan to allow *carte blanche* to the fishmonger or contractor to supply whatever happens to be plentiful. The onus of making a palatable and nutritious dish out of the material supplied must then rest with the cook. But some trouble should be taken beforehand to fix on modes of preparation adapted to every contingency. Herrings, mackerel, cod, fresh haddock, even flounders, dabs, and fresh-water fish, will all make good meals if "mixed with brains."

Fresh herrings baked are more appetising and nutritious than when boiled, or even fried. The heads, tails, and fins must be taken off, and if time allows the backbone extracted. They should then be sprinkled inside and out with pepper and salt, and laid in a deep baking-dish, with the roes at the top. They should be covered with vinegar and water, flavoured with a few cloves, and baked for one hour. They may be served hot or cold.

Herring-pie is also good. Remove heads, tails, fins, and bones, and season the fish with salt and pepper. Lay at the bottom of a pie-dish a thick layer of chopped apples and onions in equal parts. Then put a layer of herrings, and cover with another layer of the mixture, ending with herrings. Cover with mashed potato, pour in a little water, and bake.

Fish macaroni can be made with any kind of fish, which must be partly boiled, all the skin and bones removed, and broken into small pieces. To every 2 lb. of fish add two sticks of macaroni, previously boiled and cut into inch pieces; mix all well together, and put it in a fireproof dish; grate cheese over it, add a trifle of margarine and a layer of mashed potatoes. Bake for about a quarter of an hour, and serve browned.

Any small fish can be made appetising by baking them, after cleaning and trimming, in a deep dish with a sauce composed of a gill of vinegar, half a pint of melted margarine sauce made like ordinary melted butter, and two onions chopped small. They must be baked for about twenty minutes, and served with the sauce, which suffices for about 6 lb. of fish.

A plain but excellent fish pudding may be made by lining an ordinary pudding-basin with paste. The fish, carefully trimmed from bones, etc., should be cut into pieces, and seasoned with salt, pepper, and a little parsley and onion, then moistened with stock. Cover with a crust, and boil for one hour.

Other good fish dishes may be made up by the cook at will, by the expedient of covering a fireproof dish with fried onions which have been previously parboiled, adding a deep layer of partly boiled fish in small pieces, and crowning the dish with boiled rice or potatoes, or lentils or other vegetable or cereal.

Fish soup as usually prepared is rather too costly, and demands too elaborate a process to be recommended for institution use. But when plenty of fish unsuited for the table is available, such as fresh-water fish or other "small fry," it may be utilised as follows:—Take 8 or 10 lb. of smallish fish, wash them in salt and water, and stew them down with a few tomatoes, onions, leeks, and sliced carrots. Add parsley and thyme and enough water to cover all. When it is all reduced to a pulp pass it through a wide-meshed strainer and add water in proportion to the numbers. Celery and turnips previously boiled and sliced should be added. A spoonful or two of some savoury vinegar will greatly improve it. In fact, with every kind of fish soup some trouble *must* be taken over the flavouring.

Fish stock may be made at any time by boiling down the trimmings and bones of any kind of fish. The resulting liquor will be insipid and unfit for use without help, but by straining it and adding any kind of vegetable (except greens) cut in small pieces, and by flavouring it with a lemon or two, and a little Harvey or Worcester sauce, it can be made into a very agreeable soup. A more substantial soup can be made by adding to the fish stock sufficient boiled and grated potatoes to thicken it. Four pounds of potatoes will thicken two quarts of stock. A pint of milk and one or two eggs well beaten should be added. Flavour to taste, not omitting the salt.

